# When a Woman’s Heart Fails to Contain: Takotsubo Syndrome as a Gendered Collapse of Emotional Regulation

**DOI:** 10.3390/life15091431

**Published:** 2025-09-12

**Authors:** Giuseppe Marano, Enrico Romagnoli, Giuseppe Biondi-Zoccai, Gianandrea Traversi, Osvaldo Mazza, Roberto Pola, Eleonora Gaetani, Marianna Mazza

**Affiliations:** 1 Unit of Psychiatry, Fondazione Policlinico Universitario A. Gemelli IRCCS, 00168 Rome, Italy; 2 Department of Neurosciences, Università Cattolica del Sacro Cuore, 00168 Rome, Italy; 3 Department of Cardiovascular Sciences, Fondazione Policlinico Universitario A. Gemelli IRCCS, 00168 Rome, Italy; enrico.romagnoli@poilclinicogemelli.it; 4 Department of Medical-Surgical Sciences and Biotechnologies, Sapienza University, 04100 Latina, Italy; giuseppe.biondizoccai@uniroma1.it; 5 Maria Cecilia Hospital, GVM Care & Research, 48033 Cotignola, Italy; 6 Unit of Medical Genetics, Department of Laboratory Medicine, Ospedale Isola Tiberina-Gemelli Isola, 00186 Rome, Italy; gianandrea.traversi@gmail.com; 7 Spine Surgery Department, Bambino Gesù Children’s Hospital IRCCS, 00168 Rome, Italy; osvaldo.mazza1973@hotmail.it; 8 Section of Internal Medicine and Thromboembolic Diseases, Department of Internal Medicine, Fondazione Policlinico Universitario A. Gemelli IRCCS, Università Cattolica del Sacro Cuore, 00168 Rome, Italy; roberto.pola@policlinicogemelli.it; 9 Department of Translational Medicine and Surgery, Fondazione Policlinico Universitario A. Gemelli IRCCS, Università Cattolica del Sacro Cuore, 00168 Rome, Italy; eleonora.gaetani@unicatt.it; 10 Unit of Internal Medicine, Cristo Re Hospital, 00167 Rome, Italy

**Keywords:** alexithymia, emotional regulation, gender differences, psychocardiology, stress-induced cardiomyopathy, Takotsubo Syndrome

## Abstract

Background: Takotsubo Syndrome (TTS), or stress-induced cardiomyopathy, is an acute and typically reversible cardiac condition that mimics acute coronary syndrome without obstructive coronary artery disease. Predominantly affecting postmenopausal women, TTS has been increasingly recognized as a psychobiological disorder involving neuroendocrine dysregulation, autonomic imbalance, psychosocial stress, and gendered patterns of emotional regulation. This review aimed to synthesize multidisciplinary evidence to propose an integrative, gender-informed model of TTS. Methods: A narrative literature review was conducted using PubMed/MEDLINE, Scopus, and Web of Science (2000–2025) to identify clinical, neurobiological, psychosocial, and psychoanalytic studies addressing sex/gender differences, psychiatric comorbidities, and emotional regulation in TTS. Results: Evidence indicates that catecholamine surge, hypothalamic–pituitary–adrenal axis dysregulation, estrogen deficiency, and autonomic imbalance provide a biological substrate for stress-induced myocardial stunning. Psychosocial factors, such as caregiving burden, chronic stress, and alexithymia, further decrease resilience. Gendered coping scripts and unconscious symbolic processes may amplify vulnerability and influence clinical presentation. The integrative model combines biological, psychological, and social mechanisms, highlighting the predominance of emotional triggers in women and worse in-hospital outcomes in men. Conclusions: TTS should be approached as both a cardiac and affective disorder. Gender-sensitive, multidisciplinary management, including psychiatric screening, psychocardiology interventions, and psychoanalytically informed care, may improve prevention, diagnosis, and patient outcomes.

## 1. Introduction

Takotsubo Syndrome (TTS), also known as stress-induced cardiomyopathy or “broken heart syndrome”, is an acute and typically reversible cardiac condition that mimics acute coronary syndrome (ACS) through its clinical presentation but lacks obstructive coronary artery disease [1,2]. Characterized by transient left ventricular wall motion abnormalities (particularly apical ballooning), TTS often arises in the context of intense emotional or physical stress [3]. The term “takotsubo” (in Japanese “octopus pot”) was adopted by Japanese cardiologists because the acutely affected left ventricle resembles the round-bottom, narrow-neck pot used to trap octopus; hence the name Takotsubo Syndrome [4].

While the underlying pathophysiological mechanisms remain incompletely understood, a surge in catecholamines (e.g., adrenaline, noradrenaline) is widely deemed central, inducing myocardial “stunning” and microvascular dysfunction [5]. Although once considered benign, accumulating evidence suggests that TTS carries non-negligible short- and long-term risks, such as heart failure, arrhythmias, and even mortality [6].

This review pursues a gender-informed psychobiological model of TTS, integrating neuroendocrine mechanisms, psychosocial stress, emotional regulation, and a psychoanalytic perspective to elucidate why TTS disproportionately affects women, especially post-menopausal, and how gendered emotional processes may shape both vulnerability and manifestation. This review’s novelty lies in integrating cardiology, psychiatric, and psychoanalytic evidence into a gender-informed psychobiological model of TTS and outlining practical, gender-sensitive implications for screening and follow-up.

## 2. Materials and Methods

This review was conducted following the narrative review framework proposed by Sukhera [7] and aligned with the methodological guidance for non-systematic reviews [8]. The primary aim was to synthesize current knowledge on TTS through a gender-informed psychobiological lens, integrating cardiological, psychiatric, psychosocial, and psychoanalytic perspectives. A comprehensive literature search was performed in PubMed/MEDLINE, Scopus, and Web of Science from January 2000 to July 2025. The search terms included combinations of: “Takotsubo Syndrome” OR “stress cardiomyopathy”; “broken heart syndrome” OR “gender differences”; “sex differences” ”emotional regulation” OR “emotional dysregulation”; “psychocardiology” OR “psychiatric comorbidities” OR “depression” OR “anxiety”; ”psychoanalysis” OR “psychodynamic” OR “symbolic meaning”. Boolean operators (AND, OR) were used to refine searches. Reference lists of included articles and relevant reviews were manually screened to identify additional studies.

**Figure 1:**
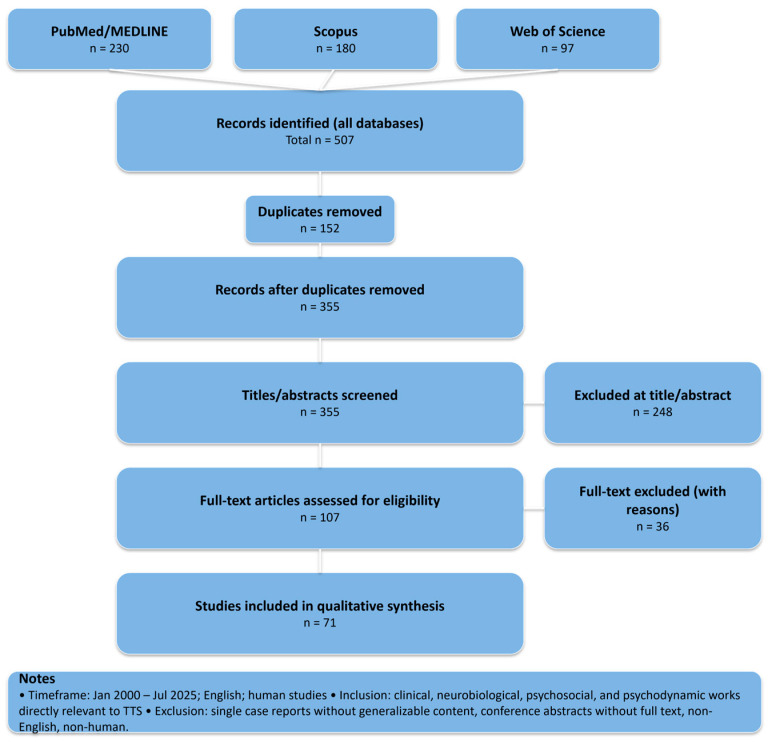
Simplified PRISMA flow for this narrative review.

We included peer-reviewed articles in English focusing on clinical studies, meta-analyses, or systematic reviews of TTS with data on sex/gender differences; studies on neuroendocrine, autonomic, psychosocial, or psychiatric aspects of TTS; research or theoretical works connecting TTS to emotional regulation or psychoanalytic constructs. Exclusion criteria were case reports without broader clinical discussion; conference abstracts without full-text availability. Data was extracted on study design, population characteristics, key findings on gender differences, neurobiological correlates, psychosocial triggers, psychiatric comorbidities, and psychoanalytic interpretations. Findings were narratively synthesized to identify recurrent patterns, gaps, and integrative models. To enhance transparency in this narrative review, we report a simplified PRISMA flow (Figure 1).

## 3. Epidemiology and Gender Differences

The global burden of TTS remains modest compared to ACS, but nontrivial. TTS accounts for approximately 1–3% of patients initially suspected of having ACS, with higher rates (up to about 5–6%) observed among female patients [9,10]. TTS represents around 0.02% of all hospital admissions [11,12].

A striking gender disparity characterizes TTS: women, particularly post-menopausal women aged roughly between 65 and 70, account for approximately 80–90% of cases [13]. This gender skew is observed consistently across Western and other regions [14,15]. Notably, in Western countries TTS is nearly nine times more common in women than in men; by contrast, in Japan the gender gap is comparatively narrower [16].

Male TTS cases, while far less frequent (~10% or somewhat less), often involve distinct demographic and prognostic characteristics. Males diagnosed with TTS tend to be younger than their female counterparts (approximately 69 vs. 71 years on average) and are more likely to have physical (rather than emotional) precipitants [16,17]. Importantly, outcomes in men are often worse: they experience more frequent in-hospital complications, severe heart failure, arrhythmias, and higher mortality [18,19]. Besides, men more often experience physical/critical-illness triggers, suggesting that non–estrogen pathways (adrenergic, microvascular, inflammatory) play a relatively greater role in male presentations, whereas postmenopausal hormonal milieu likely amplifies vulnerability in women.

These gendered differences suggest a multilayered interplay of biological (e.g., estrogen’s cardioprotective effects), endocrine, psychosocial, and perhaps diagnostic or care pathway factors that warrant further investigation.

The substantial sex and gender differences observed in TTS encompass epidemiological patterns, precipitating triggers, clinical characteristics, and prognosis. As summarized in Table 1, women represent the vast majority of cases, tend to be older at presentation, and more frequently experience emotionally triggered episodes, whereas men are more likely to present with physically triggered events and to experience more severe in-hospital outcomes. This synthesis provides a concise reference point for understanding how biological sex and gendered life experiences may shape vulnerability and clinical trajectories in TTS.

## 4. Pathophysiology: Neuroendocrine and Autonomic Mechanisms

The pathophysiology of TTS is multifactorial, involving a complex interplay between neuroendocrine stress responses, autonomic imbalance, and myocardial vulnerability. The prevailing hypothesis centers on a catecholamine surge, typically adrenaline and noradrenaline, triggered by acute emotional or physical stressors [21]. Excess catecholamines can induce direct myocardial toxicity, precipitate coronary microvascular dysfunction, and cause transient left ventricular (LV) wall-motion abnormalities [22]. The hypothalamic–pituitary–adrenal (HPA) axis plays a central role in mediating stress-induced cardiovascular responses. Dysregulation of this axis, especially in individuals with heightened sympathetic reactivity, may amplify the magnitude and duration of catecholamine release [23]. Experimental models have shown that estrogen deficiency, common in postmenopausal women, exacerbates stress-induced myocardial injury by diminishing endothelial nitric oxide availability and impairing vasodilatory capacity [24]. Autonomic imbalance, characterized by sympathetic overactivation and reduced parasympathetic tone, has been documented during the acute phase of TTS and may persist beyond recovery [25].

Inflammation has been shown to play a key role in determining outcomes in TTS patients, as well as in the early pathogenesis of the disorder. Increased cytokine levels were observed acutely in TTS, with some such as interleukin (IL)-6 and IL-10 determined to be predictors for long-term adverse events or mortality in patients [26]. Neuroimaging studies further suggest that patients with TTS display altered functional connectivity in limbic and brainstem regions involved in autonomic and emotional regulation [27]. These findings support the conceptualization of TTS as a disorder at the interface of neuroendocrinology, autonomic physiology, and emotional processing. While postmenopausal estrogen decline may contribute to the marked female predominance of TTS, via effects on endothelial function, autonomic balance, and inflammatory signaling, this does not imply an equivalent mechanism in men. In males, circulating estradiol is largely aromatase-derived from testosterone and does not exhibit an abrupt withdrawal, making a menopause-like trigger improbable. Male TTS more commonly follows physical or critical-illness stressors and appears to be driven primarily by non–estrogen-dependent pathways, including catecholamine-mediated β-adrenergic signaling adaptations, coronary microvascular/endothelial dysfunction, autonomic imbalance, and systemic inflammation/comorbidity. Accordingly, sex hormones should be viewed as one modulator within a multifactorial model rather than a universal mechanism.

Contemporary data indicate that TTS is grounded in organic pathobiology: coronary microvascular/endothelial dysfunction promotes malperfusion and microspasm; catecholamine excess induces β-adrenergic signaling adaptations that blunt contractility while engaging pro-survival cascades; autonomic imbalance (sympathetic surge/vagal withdrawal) destabilizes myocardial function; and inflammatory/immune signaling contributes to stunning and recovery dynamics. These mechanisms interact with host predisposition, for which current evidence supports a modest, likely polygenic genetic influence with gene–environment effects. Within this substrate, psychosocial stressors operate primarily as triggers and modulators rather than primary causes [21].

## 5. Psychosocial and Psychiatric Comorbidities

Psychiatric comorbidities are highly prevalent in TTS, with depression, anxiety disorders, and post-traumatic stress disorder (PTSD) being most frequently reported [28,29,30]. Epidemiological studies indicate that up to 50% of patients with TTS have a current or past psychiatric diagnosis, a prevalence significantly higher than in matched cardiac control populations [31].

Acute emotional triggers (bereavement, relational rupture, interpersonal conflict) are commonly reported in women, whereas men are more often exposed to physical triggers such as acute illness or surgery [32]. Chronic psychosocial stressors, including caregiving burden, social isolation, and financial strain, may contribute to an elevated allostatic load, reducing physiological resilience to acute stress [33]. TTS has been identified in some COVID-19 patients, associated with physical stress [34,35,36]. Notably, acute emotional precipitants are not limited to negative stress. A small but well-documented subset of cases follows intense positive emotional stimuli (‘happy heart syndrome’) after events such as the birth of a grandchild, a favorite team’s victory, or an unexpected windfall. In large registries, pleasant triggers account for ~1.5% of all TTS and ~4% of emotionally triggered TTS; these cases show a higher proportion of male patients and more frequent atypical (often midventricular) ballooning, with outcomes broadly comparable to negatively triggered events. Clinically, this underscores the need to elicit recent positive as well as negative stressors during history taking [37,38,39].

Alexithymia, the difficulty in identifying and verbalizing emotions, is overrepresented among TTS patients [40] and may predispose individuals to somatic rather than symbolic processing of distress. This is in line with psychocardiology frameworks that view TTS not only as a cardiac event but also as a psychosomatic expression of unregulated affect [41]. Consistent with the 2025 ESC Clinical Consensus Statement on Mental Health and Cardiovascular Disease, mental health assessment is now considered integral to cardiovascular care, with recommendations for routine screening and stepped-care pathways to improve outcomes [42].

## 6. Emotional Regulation and Gendered Constructs

Emotional regulation refers to the processes by which individuals influence the onset, intensity, and expression of emotions [43]. In TTS, failures in emotional regulation, whether due to dispositional traits, learned coping strategies, or situational overload, can result in physiological dysregulation that contributes to myocardial stunning [44].

Gender plays a significant role in shaping emotional regulation patterns. Women, especially in midlife and later adulthood, are often socialized to prioritize relational harmony, adopt caregiving roles, and suppress expressions of anger or distress [45]. While such strategies may be adaptive in maintaining social cohesion, they can also lead to chronic emotional containment and reduced opportunities for affective release. The predominance of TTS in postmenopausal women suggests that gendered psychosocial scripts intersect with biological vulnerability to create a “perfect storm” for stress-induced cardiac events. The symbolic dimension, captured in the colloquial term “broken heart syndrome”, highlights how cultural narratives around female emotionality may influence both the lived experience of stress and its somatic manifestations [46,47]. The multifaceted determinants of TTS can be organized into three main domains: neuroendocrine/autonomic mechanisms, psychosocial and psychiatric comorbidities, and emotional regulation with gendered constructs. Table 2 summarizes these domains, the key mechanisms involved, representative supporting evidence, and their implications for TTS pathophysiology and management.

## 7. A Psychoanalytic Perspective on Takotsubo Syndrome

While TTS has traditionally been examined within biomedical and psychophysiological frameworks, a psychoanalytic lens offers additional depth in understanding its symbolic, unconscious, and relational dimensions. From a psychodynamic perspective, the heart is not only a vital organ but also a cultural and psychic metaphor for emotional vitality, love, and resilience. The sudden, reversible “collapse” seen in TTS can be read as a somatic dramatization of overwhelming affect what Freud [48] described as the conversion of unprocessed psychic tension into bodily expression. In psychoanalytic theory, when affect cannot be adequately mentalized or symbolized, often due to early relational patterns or overwhelming stress, it may be discharged through the body [47,49]. In TTS, the acute cardiac dysfunction can be interpreted as a psychosomatic “acting-out” of uncontained emotional pain, particularly when verbal or conscious processing is inhibited. The high prevalence of alexithymia in TTS patients supports this hypothesis, suggesting that difficulties in identifying and articulating feelings may channel emotional overload into cardiac pathways. The gender skew in TTS aligns with psychoanalytic reflections on the “maternal container” function [46]. Many female patients occupy long-standing caregiving roles in which they absorb and metabolize others’ distress, often at the expense of their own psychic needs. When this containment function collapses, the body may somatically express the breakdown of emotional regulation. The popular term “broken heart syndrome” resonates with what Lacan [50] called the symbolic register: a culturally and linguistically embedded way of making sense of psychic events. The apical ballooning pattern, in this reading, is not merely a mechanical response to catecholamine excess but a corporeal “script” that conveys an unspeakable loss or rupture. This meaning-making is often unconscious, revealed indirectly through bodily crisis rather than articulated speech.

TTS often follows acute relational trauma, death of a loved one, divorce, betrayal, echoing psychoanalytic accounts of repetition compulsion, where unresolved past losses are re-enacted in the present [51]. In postmenopausal women, such events may reactivate earlier unresolved grief or attachment disruptions, magnifying the psychobiological stress response. Incorporating psychoanalytic insights into TTS care suggests that beyond standard cardiological management, therapeutic interventions should aim to restore symbolic processing of affect, re-establish safe relational containers, and address unconscious meanings attached to the illness experience. Liaison psychiatry, psychodynamic psychotherapy, or supportive-expressive therapy may help patients integrate the affective storm into their life narrative, potentially reducing recurrence risk.

## 8. An Integrative Psychobiological Model of Takotsubo Syndrome

TTS emerges at the intersection of neuroendocrine stress physiology, psychosocial context, and unconscious symbolic processes. This integrative model synthesizes three interdependent domains, biological vulnerability, emotional regulation capacity, and gendered psychosocial roles, into a unified explanatory framework.

### 8.1. Biological Vulnerability

Postmenopausal decline in estrogen, an endothelial and anti-inflammatory modulator, appears to remove a brake on sympathetic toxicity, thereby heightening susceptibility to catecholamine-mediated injury and coronary microvascular dysfunction in TTS. Animal models show that ovariectomy amplifies stress-evoked autonomic outflow and reversible left ventricular (LV) dysfunction, while estrogen supplementation attenuates central (amygdalo-hypothalamic) activation and improves cardiovascular responses, supporting a causal role for estrogen signaling along the brain–heart axis. Clinically, research emphasizes estrogen’s regulatory effects on vascular tone and oxidative stress, consistent with the marked postmenopausal predominance of TTS [52]. Beyond sex-hormone milieu, dysregulation of the stress systems, the HPA axis and the sympathetic nervous system, likely magnifies the cardiovascular impact of acute stress. Neuroimaging studies demonstrate increased amygdalar metabolic activity during the hyper-acute phase and even years before onset in individuals who later develop TTS, implicating a primed central stress network that can drive peripheral inflammatory and adrenergic responses [53,54]. Complementing this, mechanistic work indicates that high epinephrine concentrations trigger a β2-adrenoceptor coupling “switch” from Gs to Gi signaling, a protective but negatively inotropic bias that, together with β-adrenergic desensitization machinery (GRK2/β-arrestin2) upregulation in human biopsies, offers a cellular explanation for acute myocardial stunning [55]. At the coronary level, multiple invasive and non-invasive studies support a prominent role for microvascular dysfunction: elevated Index of Microcirculatory Resistance (IMR)/reduced coronary flow reserve (CFR) in the acute setting, apical-predominant impairment that correlates with LV dysfunction, and diffuse myocardial edema on Cardiac (Cardiovascular) Magnetic Resonance (CMR) without infarction, findings that map onto catecholamine toxicity, endothelial dysregulation, and vasomotor instability [56]. These data reinforce that TTS emerges when heightened sympathetic/HPA reactivity meets a vulnerable vascular–myocardial substrate in a postmenopausal context. Finally, genetic susceptibility remains plausible but unresolved. Small candidate-gene studies suggest links between β1/β2-adrenergic receptor variants and TTS phenotypes, whereas larger cohorts have reported neutral results; recent reviews conclude that any inherited contribution is likely modest and polygenic, interacting with endocrine and environmental stressors rather than operating as a single-gene driver [57].

### 8.2. Emotional Regulation Capacity

Effective regulation presupposes the ability to identify, symbolize, and communicate affective states (the “extended process model” of emotion regulation). Deficits at the identification/labeling stage push individuals toward late, response-focused strategies (e.g., suppression) that carry higher physiological costs [58]. In TTS, data suggests impairments in emotional competencies and alexithymic traits. Compared with matched controls and AMI comparators, TTS patients, especially those with emotion-triggered events, show lower emotional intelligence and greater emotional-processing deficits independent of depressive symptoms; case-controlled work also notes elevated alexithymia in TTS relative to norms. Single-case twin evidence further links the TTS phenotype to higher anxiety, vital exhaustion, social inhibition, and alexithymia, underscoring a vulnerability profile in which emotions are poorly mentalized and more likely to be “discharged” somatically [59]. Mechanistically, alexithymia is associated with atypical interoception and insula-mediated awareness of bodily signals, features tied to heightened subjective stress and altered autonomic control. Meta-analytic and neurobiological studies relate alexithymia to deficits in emotional awareness and interoceptive processing (insula/anterior cingulate), while experimental work shows facet-specific links to autonomic reactivity during social stress [60,61]. These identification-stage deficits matter clinically because labeling emotion appears to down-regulate amygdala activity via prefrontal pathways, whereas unlabeled affect remains more somatically expressed, plausibly amplifying the neurocardiac impact of acute stress in susceptible patients [62]. By contrast, expressive suppression, a common fallback when labeling/meaning-making fails, raises cardiovascular load. Laboratory and ambulatory studies show that suppression increases sympathetic activation and stress-induced cardiovascular reactivity, whereas cognitive reappraisal is generally less costly. Suppression also disrupts social communication, potentially removing interpersonal buffering during stress [63,64]. These dynamics coincide with clinical observations in TTS of high trait anxiety/psychological distress and a tendency toward somatic channels of affect [65].

Sociocultural factors can reinforce this pathway. Developmental work documents small but reliable gender differences in emotional expressivity and socialization practices (e.g., expectations to remain composed/caregiving), patterns that may normalize suppression and reduce help-seeking until a severe stressor precipitates dysregulation [66]. Finally, measurement caveats are warranted: widely used alexithymia scales (e.g., Toronto Alexythimia Scale-TAS-20) partially track general distress, so elevations in acute TTS cohorts should be interpreted alongside convergent markers (interoception indices, clinician-rated processing measures) [67].

### 8.3. Gendered Psychosocial Roles

TTS’s striking predominance in women points to sociocultural patterning of stress exposure and response [68]. Conversely, positive-emotion precipitants, though rare, are increasingly recognized and may present with somewhat different phenotypic features (e.g., non-apical ballooning) and a modestly higher male proportion [69,70].

Women perform the vast majority of unpaid caregiving worldwide and shoulder disproportionate “emotional labor,” both of which are linked to higher perceived stress; among midlife “sandwich-generation” carers, financial/emotional overload and worse self-rated health are consistently reported [71]. Within a gendered script that prioritizes composure and self-sacrifice, help-seeking may be deferred and distress suppressed, further removing interpersonal buffering during adversity [72]. Over time, these role demands accumulate as allostatic load, the biologic “wear and tear” of chronic stress, thereby priming neuroendocrine and inflammatory systems for dysregulated responses when a major stressor strikes [73]. Consistent with this pathway, large registries and reviews document bereavement, interpersonal conflict, and domestic abuse among canonical precipitants of TTS [74]. Taken together, gendered caregiving and relational roles can amplify chronic stress load and shape the type of acute trigger encountered, creating conditions under which an acute event (e.g., loss or rupture) precipitates the neurocardiac cascade characteristic of TTS.

### 8.4. The Unconscious and Symbolic Layer

A psychoanalytic perspective enriches the model by framing Takotsubo’s acute collapse as a failure of symbolization under extreme affective load. In Bion’s terms, when the containing “alpha function” breaks down, raw, unmentalized emotional “beta-elements” are expelled somatically rather than transformed into thinkable experience, so the “broken heart” metaphor can become a literal bodily event [46]. McDougall’s classic psychosomatic formulation similarly reads such crises as “theatres of the body”, where psychic conflict is enacted in corporeal form when representational pathways are blocked [47]. Complementing this, the Paris psychosomatic school described operative/operational thinking, a concrete, low-symbolic mode of functioning that channels excitation toward behavior or somatic outlets when fantasy and reflection are impoverished, mapping closely onto alexithymic styles observed around TTS [49]. Developmental-clinical work on mentalization adds a bridge to contemporary affect-regulation science: when the capacity to identify and hold mind–body states in symbolic form is compromised, individuals rely on late, response-focused strategies (e.g., suppression) that are physiologically costly [75]. Converging neurocardiac evidence supports this mind–heart link: individuals who later develop TTS show heightened amygdalar activity and altered brain metabolic patterns months to years before onset, suggesting a primed threat-network whose output can amplify autonomic and inflammatory cascades during acute stress [76]. Clinically, this rationale underpins the inclusion of psychotherapeutic engagement (supportive/expressive or mentalization-focused work, often alongside treatment of anxiety/depression) in recovery and relapse prevention plans, an area now being tested in dedicated psychosocial-support studies, amid registry data showing that no somatic therapy reliably prevents recurrence [77].

Although Takotsubo-specific randomized trials of mindfulness are lacking, mind–body approaches are being tested (e.g., deep-breathing trials), and contemporary reviews note MBIs as promising adjuncts. In broader cardiovascular populations, mindfulness-based interventions (including Mindfulness-Based Stress Reduction and Mindfulness-Based Cognitive Therapy) reduce perceived stress, improve blood pressure and selected cardiovascular health metrics, and enhance vagal activity indexed by heart rate variability (HRV), suggesting biologically plausible pathways to mitigate stress reactivity relevant to TTS [78,79].

**Figure 2:**
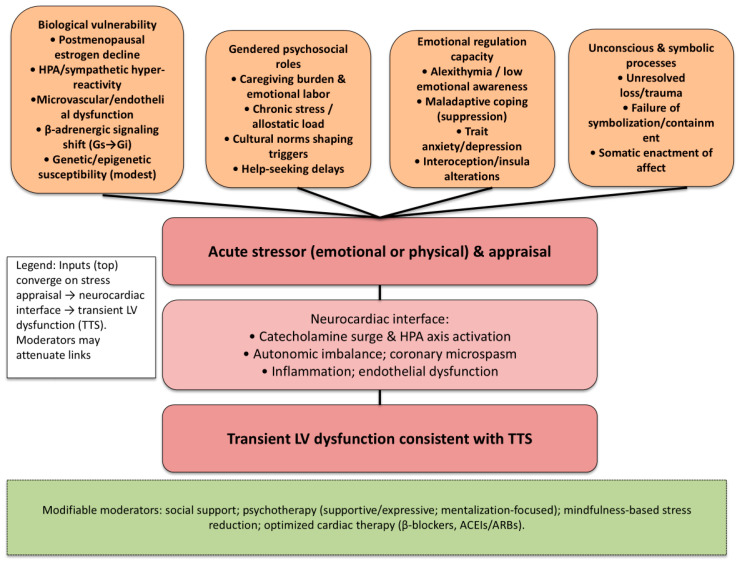
Integrative psychobiological model of Takotsubo Syndrome (TTS). Note: Upstream inputs: (1) biological vulnerability (postmenopausal estrogen decline; HPA/sympathetic hyper-reactivity; microvascular/endothelial dysfunction; β-adrenergic signaling shift; modest genetic/epigenetic susceptibility), (2) gendered psychosocial roles (caregiving burden, chronic stress/allostatic load, cultural norms, help-seeking delays), (3) emotional-regulation capacity (alexithymia, suppression, trait anxiety/depression, interoception/insula alterations), and (4) unconscious/symbolic processes (unresolved loss/trauma, failure of symbolization/containment, somatic enactment) converge on acute stressor appraisal. Directional arrows indicate the flow from appraisal to the neurocardiac interface (catecholamine surge and HPA activation; autonomic imbalance; coronary microspasm; inflammation/endothelial dysfunction), ultimately resulting in transient LV dysfunction typical of TTS. A dashed box highlights modifiable moderators (social support; psychotherapy—supportive/expressive, mentalization-focused; mindfulness-based stress reduction; optimized cardiac therapy with β-blockers and ACEIs/ARBs) that may attenuate multiple links along the pathway. Abbreviations: TTS, Takotsubo syndrome; HPA, hypothalamic–pituitary–adrenal (axis); LV, left ventricular; β-adrenergic, beta-adrenergic; β-blockers, beta-adrenergic blockers; ACEIs, angiotensin-converting enzyme inhibitors; ARBs, angiotensin II receptor blockers; Gs/Gi, stimulatory/inhibitory G-protein.

These layers do not act in isolation. Biological predisposition shapes the physiological stress response; emotional regulation skills determine whether affect is metabolized or somatized; gendered roles influence exposure to chronic stress and containment demands; and unconscious symbolic processes shape the form of the bodily symptom. The acute stressor, emotional or physical, functions as the final trigger in this multidimensional vulnerability network. In Figure 2 it is proposed an integrative psychobiological model of TTS, showing the interaction between biological vulnerability, emotional regulation capacity, gendered psychosocial roles, and unconscious symbolic processes, culminating in acute cardiac dysfunction. The figure depicts how distal vulnerabilities and proximal precipitants converge on acute cardiac dysfunction. Biological vulnerability (including genetic predisposition, post-menopausal estrogen depletion, autonomic dysregulation, and microvascular/endothelial dysfunction) sensitizes the organism to stress. At the same time, gendered psychosocial roles (caregiving burden, social expectations, work–family strain, exposure to interpersonal stress) contribute to chronic allostatic load and shape the meanings assigned to adversity. Emotional regulation capacity (e.g., alexithymia, maladaptive coping, intolerance of uncertainty, trait anxiety/depression) and unconscious symbolic processes (unresolved loss, dependence–control conflicts, traumatic memory activation, somatization within attachment schemas) bias the appraisal of threat. An acute stressor, external or internal, undergoes stress appraisal, which gates activation of a neurocardiac interface characterized by sympathetic “catecholamine surge,” HPA axis activation with cortisol release, vascular inflammation and endothelial dysfunction, coronary microspasm, and further autonomic imbalance. The downstream effect is transient acute cardiac dysfunction consistent with TTS. The model also highlights modifiable moderators (social support, psychotherapy, mindfulness-based strategies, beta-blockers, and renin-angiotensin system agents (ACE inhibitors [ACEIs] and angiotensin receptor blockers [ARBs]), which can attenuate appraisal, blunt neurohumoral overactivation, and facilitate recovery. Overall, the diagram emphasizes that Takotsubo emerges from the dynamic interplay of biological susceptibility, psychosocial context, and symbolic/emotional processing, with identifiable intervention points across the pathway.

## 9. Prognosis by Psychological Status and Risk of Recurrence

Although TTS is typically reversible at the ventricular level, contemporary data indicate that the overall prognosis is not benign. Long-term mortality and major adverse events can approximate those seen in acute coronary syndromes. Recent summaries report non-trivial mortality during follow-up underscoring the need for structured follow-up [1,22]. It has been described a high burden of psychiatric comorbidity in TTS, particularly mood and anxiety disorders, PTSD symptoms, and markers of impaired emotion regulation (e.g., alexithymia), when compared with reference populations or acute myocardial infarction controls. While effect sizes and instruments vary, these findings are reproducible across cohorts and support routine psychosocial screening during and after the index event [31]. Recurrence after an index TTS episode is uncommon but clinically important, ranging from ~1–6% across many cohorts to ~9–10% with longer follow-up; some series report ~4% within the first year. Recurrences have been documented from 30 days to nearly 10 years after the index event [41,68]. Importantly, recurrence has been associated with higher short-term cardiovascular risk; one recent analysis reported ~5.9-fold higher 30-day CV mortality among patients with recurrence versus those without, arguing for closer surveillance in this subgroup [10].

Evidence on the hypothesis that psychological status may predict recurrencies is mixed. Some studies suggest that pre-existing psychiatric illness is associated with a higher risk of recurrent TTS, whereas others find no clear differences in standard psychosocial measures between patients with and without recurrence. Methodological differences (sample size, timing, measurement tools, and confounding by physical triggers/comorbidities) likely contribute to the heterogeneity [31,33,65]. In practice, it is recommended systematic screening during admission and follow-up for depression/anxiety and trauma-related symptoms; referral to psychocardiology or integrated cardio-behavioral care where available; patient education on stress-management and trigger awareness; and identification of high-risk profiles (male sex, physical triggers, significant comorbidity, or prior recurrence) for closer monitoring. While mind–body and psychotherapeutic interventions improve stress and functional capacity in broader cardiovascular populations, TTS-specific randomized evidence remains limited; such approaches should be framed as adjunctive, with transparent discussion of the evidence gaps [68,71,73]. The non-benign prognosis is consistent with the organic severity of TTS and the weight of trigger profiles/comorbidities, reinforcing that psychosocial factors alone cannot explain outcomes.

## 10. Clinical Implications

The integrative psychobiological model of TTS outlined in this review highlights the need for diagnostic, preventive, and therapeutic strategies that address not only cardiovascular function but also psychological, social, and symbolic dimensions of patient care. Given the strong association between TTS and psychiatric comorbidities, particularly depression, anxiety, and alexithymia, systematic mental health screening should be incorporated into cardiology settings. Standardized tools such as the Hospital Anxiety and Depression Scale (HADS) or the Toronto Alexithymia Scale (TAS) can be applied during hospitalization and follow-up to identify high-risk profiles [80]. The predominance of TTS among postmenopausal women warrants gender-tailored screening protocols. Beyond traditional cardiac risk factors, assessments should consider psychosocial stress load, caregiving burden, and life-stage transitions [81]. Clinicians should be alert to the cumulative allostatic load in female patients, which may amplify vulnerability to acute stress responses. Multidisciplinary care models, combining cardiologists, psychiatrists, psychologists, and social workers, are essential for addressing the multidimensional nature of TTS. Psychoeducation about the mind-heart connection, stress management techniques, and brief psychotherapeutic interventions may reduce recurrence risk [82]. Interventions such as mindfulness-based stress reduction, cognitive-behavioral therapy, or supportive-expressive psychotherapy can enhance emotional regulation skills and improve quality of life. For selected patients, particularly those with recurrent episodes, high symbolic resonance of illness, or unresolved grief, psychodynamic or psychoanalytically informed therapy may help explore unconscious meanings, integrate affective experiences, and restore symbolic processing [83]. Such approaches may be particularly valuable for individuals with histories of relational trauma or chronic emotional suppression.

Post-discharge programs should integrate physical and psychological rehabilitation. Structured cardiac rehabilitation programs can be adapted to include stress resilience training and gender-specific education. Longitudinal follow-up should monitor both cardiac and emotional health, as TTS survivors may remain vulnerable to future episodes and ongoing psychological distress [22].

Despite growing recognition of TTS as a complex psychobiological disorder, several critical gaps remain in our understanding and management of the condition. Most available data derive from retrospective analyses or short-term prospective studies. Longitudinal research with extended follow-up is needed to clarify long-term cardiovascular and psychological outcomes, recurrence patterns, and potential chronic sequelae [20]. Mechanistic studies integrating neuroimaging, neuroendocrine profiling, and cardiac functional assessments could elucidate how stress reactivity translates into myocardial stunning in susceptible individuals. The pronounced gender disparity in TTS incidence underscores the need for sex-disaggregated analyses that distinguish between biological sex effects (e.g., estrogen depletion, autonomic function) and gender-related psychosocial determinants (e.g., caregiving roles, societal norms). Multiomic approaches may help identify genetic and epigenetic factors modulating risk in women and men [84].

Although psychiatric comorbidities are common in TTS, mental health assessment remains inconsistent across clinical settings. Research should aim to validate standardized screening tools for emotional regulation, alexithymia, and trauma history in cardiology practice. Such tools could enable early identification of high-risk patients and guide targeted interventions [85].

Evidence for the efficacy of psychocardiology interventions in TTS is limited. Randomized controlled trials testing multimodal approaches, combining optimized cardiac care, psychological therapy, and stress management, are needed to assess impact on recurrence rates, functional recovery, and quality of life. While the role of unconscious processes and symbolic meaning in TTS has been conceptually discussed, empirical investigation is lacking. Mixed-methods studies integrating psychodynamic assessment with physiological monitoring could help quantify how unconscious affective states influence cardiovascular outcomes. In the absence of TTS-specific randomized trials, resilience-building, psychotherapy, and mind–body strategies should be presented as adjunctive options to support symptom relief (e.g., anxiety, sleep, perceived stress), coping, and adherence within multidisciplinary follow-up. Their effects on TTS incidence, recurrence, or mortality remain uncertain, and no causal inferences should be drawn for TTS outcomes at this time. Shared decision-making should include a transparent discussion of potential benefits for well-being versus the current lack of TTS-targeted outcome data.

Given variations in TTS incidence and gender distribution across cultures, comparative research could uncover sociocultural factors that shape stress exposure, emotional expression, and help-seeking behaviors. This may inform culturally sensitive prevention and treatment strategies [86].

ESC 2025 Clinical Consensus Statement recommends to integrate brief mental-health screening into TTS workflows; when TTS is suspected or confirmed, to ask systematically about recent stressors and symptoms, and consider PTSD screening as part of risk assessment, to use a stepped-care approach (education/self-management → psychological therapies → pharmacotherapy when indicated) and coordinate care within a multidisciplinary “Psycho-Cardio” team [42].

## 11. Conclusions

TTS exemplifies the intimate and bidirectional interplay between the heart and the mind. Far from being a purely cardiac phenomenon, TTS reflects a convergence of biological vulnerability, emotional regulation capacity, gendered psychosocial roles, and unconscious symbolic processes. This integrative psychobiological perspective underscores that acute cardiac dysfunction in TTS is not merely a reaction to stress but the endpoint of a complex, multidimensional vulnerability network.

Recognizing TTS as a condition shaped by both physiological and psychological determinants has important implications for prevention, diagnosis, and care. Gender-sensitive screening, systematic assessment of psychiatric comorbidities, and the integration of psychocardiology into standard cardiac care can enhance patient outcomes and potentially reduce recurrence. Furthermore, psychoanalytic insights into the symbolic meaning of illness and the role of unprocessed affect offer additional depth to patient-centered interventions. Evidence for resilience/stress-management approaches in TTS is indirect, largely extrapolated from broader cardiovascular populations and small observational TTS cohorts with heterogeneous measures and follow-up. Accordingly, any implication of outcome modification in TTS would be premature.

TTS should be approached as a multifactorial neurocardiac syndrome in which organic mechanisms (microvascular, adrenergic, autonomic, inflammatory, and modest genetic predisposition) are central, and psychosocial factors act as clinically relevant modulators and triggers, informing screening and follow-up but not supplanting disease biology.

Future research should aim to validate this integrative model, exploring sex-specific mechanisms, standardized assessment protocols, and culturally tailored interventions. Pragmatic, multicenter randomized controlled trials in TTS are needed to test defined resilience/stress-management programs against usual care, with prespecified endpoints (e.g., recurrence, major adverse cardiovascular events, readmissions, validated psychometrics), adequate sample size, and standardized follow-up to determine whether these interventions influence clinical outcomes beyond symptom relief.

By bridging cardiology, psychiatry, psychology, and psychoanalysis, the management of TTS can evolve toward a truly interdisciplinary paradigm, one that addresses not only the stunned myocardium but also the distressed, and sometimes “broken,” human heart.

## Figures and Tables

**Table 1:** Summary of Sex/Gender Differences in Takotsubo Syndrome.

Domain	Findings in Women	Findings in Men	References
Epidemiology	~80–90% of cases; mean age 65–70; predominance in postmenopausal women	~10–20% of cases; mean age slightly younger (~69 years)	Galli et al., 2019 [9]; Giubilato et al., 2024 [16]
Common Triggers	More often emotional (bereavement, relational rupture)	More often physical (acute illness, surgery, trauma)	Pelliccia et al., 2022 [20]; Arcari et al., 2022 [17]
Clinical Features	Similar chest pain and dyspnea presentation; higher prevalence of psychiatric comorbidity	Similar ACS-like symptoms; less psychiatric comorbidity but more severe hemodynamic compromise	Salamanca & Alfonso, 2023 [2]; Murakami, 2022 [13]
Prognosis	Slightly lower in-hospital mortality (~5.5%); better long-term outcomes	Higher in-hospital mortality (~11.2%); more frequent in-hospital complications (shock, arrhythmias)	Murakami, 2021 [18]

Note: Sex-specific pattern: emotional triggers more frequently reported in women; physical triggers relatively more common in men. Estimates vary across cohorts/registries. Abbreviations: ACS, Acute Coronary Syndrome.

**Table 2:** Summary of Key Domains in the Psychobiological Model of TTS.

Domain	Key Mechanisms/Factors	Representative Evidence	Implications for TTS
Neuroendocrine & Autonomic	Catecholamine surge; HPA axis dysregulation; sympathetic overactivation; reduced parasympathetic tone; estrogen deficiency	Templin et al., 2015 [22]; Ueyama et al., 2003 [24]	Acute myocardial stunning, microvascular dysfunction, and wall-motion abnormalities triggered by stress
Psychosocial & Psychiatric	Depression, anxiety, PTSD, alexithymia; acute emotional or physical triggers; chronic stress/allostatic load	Lisci et al., 2025 [29]; Compare et al., 2018 [40]; Wallström et al., 2016 [33]	Lower resilience to stress; increased susceptibility to TTS episodes and recurrence
Emotional Regulation & Gendered Constructs	Difficulty identifying/expressing emotions; suppression of distress; gendered coping roles; cultural norms about female emotionality	Klein et al., 2020 [44]; McDougall, 1989 [47]	Intersection of psychosocial scripts and biological vulnerability creates heightened risk in postmenopausal women

Abbreviations: HPA, Hypothalamic–Pituitary–Adrenal axis; PTSD, Post-Traumatic Stress Disorder; TTS, Takotsubo Syndrome.

## Data Availability

No new data was created.
